# IDO1 Expression Increased After Neoadjuvant Therapy Predicts Poor Pathologic Response and Prognosis in Esophageal Squamous Cell Carcinoma

**DOI:** 10.3389/fonc.2020.01099

**Published:** 2020-07-07

**Authors:** Ruidi Jiao, Xiaoli Zheng, Yanan Sun, Zhuo Feng, Shuai Song, Hong Ge

**Affiliations:** ^1^Department of Radiation Oncology, The Affiliated Cancer Hospital of Zhengzhou University, Henan Tumor Hospital, Zhengzhou, China; ^2^The School of Basic Medical Sciences, Zhengzhou University, Zhengzhou, China

**Keywords:** ESCC, IDO1, CD8, neoadjuvant therapy, prognosis

## Abstract

Indoleamine 2,3-dioxygenase (IDO1) plays an important role in tumor immune evasion. In this study, we investigated the changes of tumor IDO1 expression and CD8+ tumor-infiltrating lymphocytes (TILs) status in tumor microenvironment (TME) after neoadjuvant chemoradiotherapy (NCRT) or neoadjuvant chemotherapy (NCT) in esophageal squamous cell carcinoma (ESCC), respectively. Moreover, the potential predictive value of the changes of tumor IDO1 expression and CD8+TILs status on pathologic response and clinical outcome was further evaluated. By matching propensity scores in 295 patients, a total of 85 ESCC patients with neoadjuvant therapy followed by surgery were recruited, including 17 patients with NCRT and 68 patients with NCT. Tumor IDO1 expression and CD8+TILs within TME in paired specimens were evaluated by immunohistochemistry, and the changes of tumor IDO1 expression and CD8+TILs between the paired specimens were estimated. Tumor IDO1 expression significantly increased from baseline to postoperative tumor tissue after NCT (*p* = 0.002), whereas no significant difference was detected after NCRT (*p* = 0.44). The density of CD8+TILs in the tumor-invasive margin increased significantly after neoadjuvant therapy, and there was no significant difference in density changes of CD8+TILs between the NCRT and NCT groups (*p* = 0.118). Upregulation of tumor IDO1 expression after neoadjuvant therapy was associated with poor pathologic response (*p* = 0.002). Lastly, multivariate Cox analysis showed that IDO1-rise patients after neoadjuvant therapy were related to poor prognosis (*p* = 0.047). These results indicated that chemotherapy could promote tumor IDO1 expression, and the increased tumor IDO1 expression after neoadjuvant therapy predicted poor pathologic response and prognosis in ESCC.

## Introduction

Esophageal cancer is one of the most common cancers with higher morbidity and mortality in the world, leading the eighth and sixth, respectively (1). Esophageal squamous cell carcinoma (ESCC) and esophageal adenocarcinoma are the two major histological types of esophageal cancer, and its incidences are closely relevant to geographical location (2). In China, esophageal cancer, mainly ESCC, is the 3rd most common cancer and the 4th leading cause of cancer-related deaths, which is a serious threat to health (3). Surgery remains the primary treatment for resectable ESCC, and neoadjuvant chemoradiotherapy (NCRT) or chemotherapy (NCT) is utilized to downstage and improve local control and clinical outcomes (4, 5). Although some studies have emphasized NCRT achieved a significantly better complete pathologic response rate and a better survival benefit compared with NCT, the pathological complete response rate was <40% (6–8).

Currently, immunotherapy has caused widespread concern and there are increasing researches on tumor immune microenvironment. Mounting evidence indicated that the tumor expression of programmed death-ligand 1 (PD-L1) played an important role in tumor immune evasion, which was associated with poor pathological response and worse prognosis in esophageal cancer (9–12). Besides, the indoleamine 2,3-dioxygenase (IDO1), a rate-limiting enzyme, is also discussed as a potential key target on tumor immune evasion and generates immunosuppressive metabolites through oxidizing tryptophan into kynurenine (13). IDO1 activation can inhibit the function of cytotoxic T cells by promoting cell cycle stagnation and apoptosis, and downregulate the natural killer cell receptors (14).Furthermore, IDO1 can also boost the activity of regulatory T cells to engage the tumor immune escape mechanism (13). Recently, many studies have researched the IDO1 expression status of tumor cells and the density of CD8+ tumor-infiltrating lymphocytes (TILs) to predict the clinical outcome of esophageal cancer. Kiyozumi et al. manifested that patients with IDO1-positive and CD8+TIL-low expression tumors had poor prognosis compared with other patients with surgically resected esophageal cancer (15). Zhou et al. indicated that the tumor IDO1 expression before NCRT was associated with poor pathologic response and recurrence in ESCC (9). Additionally, several studies also showed that high IDO1 expression in tumor was correlated with worse overall survival (OS) in esophageal cancer (16, 17). Interestingly, Lim et al. discovered that PD-L1 expression significantly increased after NCRT and reduced after NCT in ESCC (18). Similarly, Chen et al. also found that NCRT could increase tumor PD-L1 expression and CD8+TILs counts in tumor microenvironment (TME) in rectal cancer (19). However, the influence of neoadjuvant therapy on IDO1 expression and CD8+TILs status in ESCC remains unknown.

In this study, we aimed to investigate whether the tumor IDO1 expression and CD8+TILs status would change after neoadjuvant therapy in ESCC, simultaneously compare the changes of tumor IDO1 expression and CD8+TILs status after NCRT and NCT, and evaluate the potential predictive role of the changes of tumor IDO1 expression and CD8+TILs status after neoadjuvant therapy on pathologic response and clinical outcome.

## Materials and Methods

### Patients and Tumor Specimens

We retrospectively recruited 295 ESCC patients who received NCRT or NCT followed by curative surgery between October 2012 and November 2018 at The Affiliated Cancer Hospital of Zhengzhou University for this study. Endoscopic biopsies at the time of diagnosis and surgical specimens after neoadjuvant therapy for all patients were available. Among these patients, 278 patients received NCT while 17 patients received NCRT. Clinical tumor staging and pathologic tumor stage were based on the American Joint Committee on Cancer Staging Manual 6th and 8th editions, respectively (20, 21). Approval for this study was obtained from the ethic committees of our institution.

In the NCT group, all patients underwent 2 cycles induction chemotherapy, including 230 patients treated with the chemotherapy regimen of paclitaxel and cisplatin every three weeks (150 mg/m^2^/day paclitaxel on days 1; 75 mg/ m^2^ cisplatin on days 1-5), and 48 patients treated with the chemotherapy regimen of docetaxel and cisplatin every three weeks (75 mg/m^2^/day docetaxel on days 1; 75 mg/m^2^ cisplatin on days 1-5). In the NCRT group, 9 patients underwent the same regimen of paclitaxel/cisplatin induction chemotherapy with 2 cycles; 8 patients were treated with the induction chemotherapy regimen of paclitaxel and nedaplatin every week with 4 cycles (50 mg/m^2^/day paclitaxel on days 1; 30 mg/m^2^/day nedaplatin on days 1). During treated with induction chemotherapy, patients in the NCRT group also concurrently received a total of 36-40Gy/20 fraction radiation therapy using three-dimensional conformal radiotherapy or intensity-modulated radiotherapy. After having finished neoadjuvant therapy, all patients received radical resection treatment.

Patients were followed up every 1-3months during the first two years, and thereafter every 6 months until death or September 1, 2019, whichever came first. Disease-free survival (DFS) was defined as the time from the date of surgery to the date of disease recurrence or death. Overall survival (OS) was defined as the time from the date of surgery until the date of death. Tumor regression grade (TRG) in postoperative specimens stained with hematoxylin and eosin was assessed based on the estimated percentage of vital residual tumor cells (VRTCs) by two pathologists who were unaware of other clinical data: I, >50% VRTCs; II, 10%-50% VRTCs; II, nearly complete response with <10% VRTCs; and IV, complete response (22). TRG I and II were considered as poor response, while TRG III and IV were considered as good response.

### IDO1 Immunohistochemistry Staining

Formalin-fixed, paraffin-embedded endoscopic biopsies and surgical specimens of all patients were obtained, and sliced into 3-μm sections for immunohistochemistry. After deparaffinizing the tissue sections, antigen was retrieved in Ethylene diamine tetra-acetic acid buffer (pH 9.0) using a pressure cooker at 130°C for 45 s, then incubated with anti-IDO1 rabbit polyclonal antibody (1:200; 13268-1-AP; Proteintech lab, Chicago, IL, USA) overnight at 4°C. Secondary antibody, goat anti-mouse, or goat anti-rabbit/horse radish peroxidase (HRP) (Gene Tech Company Limited, Shanghai, China), was subsequently added to the slides and followed by counterstain with hematoxylin. We valued the intensity of IDO1 expression in the tumor cells (0, no expression; 1, weak expression; 2, moderate expression; or 3, strong expression) and the proportion of stained cells (0–100%). The IDO1 expression scores were calculated by multiplying the intensity of expression and proportion of stained cells. Appropriate negative and positive controls were included in each immunohistochemical analysis. All immunohistochemically stained slides were analyzed by two pathologists who were blinded to other clinical data.

### CD8 Immunostaining

After deparaffinization and antigen retrieval, the slides were incubated with CD8 mouse monoclonal antibody (1:1000; 66868-1-Ig, Proteintech lab, Chicago, IL, USA) overnight at 4°C, subsequently incubated with Secondary antibody, goat anti-mouse, or goat anti-rabbit/horse radish peroxidase (HRP) (Gene Tech Company Limited, Shanghai, China), and then counterstained with hematoxylin. We counted the density of CD8+ TILs within 3 tumor-invasive marginal areas using microscopy at 200 × magnification, calculated and recorded the average density as the CD8+ TILs intensity in this specimen.

### Statistical Analysis

Statistical analyses were performed with SPSS 24.0 software package (SPSS Institute, Chicago, IL, USA) and JMP version 10 software package (SAS Institute, Cary, NC). We considered a *P* value (two-sided) <0.05 as statistically significant. The Chi-square or Fisher exact test was used to compare the clinicopathological characteristics of patients between NCRT group and NCT group. Wilcoxon rank test was used to evaluate the intra-group changes; and the differences in changes of IDO1 expression score and CD8+TILs status between two groups were evaluated by Mann-Whitney *U*-test. We plotted the receiver-operating characteristic (ROC) curves for the sensitivity and specificity of the changes of IDO1 expression and the density changes of CD8+ TILs in predicting death within 24 months in 82 ESCC patients, respectively. Survival curves were generated by using the Kaplan-Meier method, and compared by the log-rank test. Cox-regression model was used for univariate and multivariate survival analyses.

For purpose of weakening the effects of unbalanced covariates between NCRT group and NCT group, a propensity score (PS) was calculated to create well-balanced groups. The PS for 295 patients was approximated using a multivariable logistic regression model that included the following variables: age, sex, ECOG performance status, tobacco use, alcohol use, comorbidity, tumor location, clinical stage, cT stage, and cN stage. According to the propensity score, 17 patients in the NCRT group were matched 1:4 to patients in the NCT group using the global optimum method (23).

## Results

### Patient Characteristics

A total of 295 ESCC patients who received neoadjuvant therapy and followed by surgery were collected, including 17 patients with NCRT and 278 patients with NCT. Clinical characteristics were not significantly different between the NCRT group and the NCT group (Table 1). After propensity matching, 17 patients in the NCRT group and 68 patients in the NCT group were included in the analysis, and there was an expected balance between the two groups in age, sex, ECOG performance status, tobacco use, alcohol use, comorbidity, tumor location, clinical stage, cT stage, and cN stage (Table 1).

**Table 1:** Clinical characteristics of the patients before and after propensity score matching.

	**Overall *N* = 295 (%)**	**Before Matching**				**After Matching**					
		**Group NCT**		**Group NCRT**		***P***	**Group NCT**		**Group NCRT**		***P***
		***N* = 278**	**%**	***N* = 17**	**%**		***N* = 68**	**%**	***N* = 17**	**%**	
Age						0.183					0.722
<70 years	263 (89.2)	250	89.9	13	76.5		57	83.8	13	76.5	
≥70 years	32 (10.8)	28	10.1	4	23.5		11	16.2	4	23.5	
Sex						0.593					0.638
Male	216 (73.2)	205	73.7	11	64.7		48	70.6	11	64.7	
Female	79 (26.8)	73	26.3	6	35.3		20	29.4	6	35.3	
Performance status						0.800					1.000
0	50 (16.9)	48	17.3	2	11.8		9	13.2	2	11.8	
1	245 (83.1)	230	82.7	15	88.2		59	86.8	15	88.2	
Tobacco use						0.251					0.823
Yes	144 (48.8)	138	49.6	6	35.3		26	38.2	6	35.3	
No	151 (51.2)	140	50.4	11	64.7		42	61.8	11	64.7	
Alcohol use						0.521					1.000
Yes	117 (39.7)	109	39.2	8	47.1		32	47.1	8	47.1	
No	178 (60.3)	169	60.8	9	52.9		36	52.9	9	52.9	
Comorbidity						0.775					1.000
Absent	217 (73.6)	205	73.7	12	70.6		48	70.6	12	70.6	
Present	78 (26.4)	73	26.3	5	29.4		20	29.4	5	29.4	
Tumor location						0.709					0.714
Upper/middle	214 (72.4)	201	72.3	13	76.5		49	72.1	13	76.5	
Distal	81 (27.5)	77	27.7	4	23.5		19	27.9	4	23.5	
Clinical stage						0.620					0.737
II	105 (35.6)	98	35.3	7	41.2		25	36.8	7	41.2	
III/IV	190 (64.4)	180	64.7	10	58.8		43	63.2	10	58.8	
cT stage						1.000					0.938
II/III	248 (84.1)	234	84.2	14	82.4		59	86.8	14	82.4	
IV	47 (15.9)	44	15.8	3	17.6		9	13.2	3	17.6	
cN stage						0.514					0.493
N0	100 (33.9)	93	33.5	7	41.2		22	32.4	7	41.2	
N1	195 (66.1)	185	66.5	10	58.8		46	67.6	10	58.8	

Overall, 85 patients with biopsies tissue samples and surgical specimens were analyzed in our study. The median age was 59 years (range, 46–75), and more than two-thirds of the patients were men. 84.7% of patients had clinical stage II/III diseases. The median interval between the end of neoadjuvant therapy and esophagectomy was 4.0 weeks (range, 2.0-9.6) with 3.1 weeks (range, 2.0-6.6) in the NCRT group and 4.3 weeks (range, 2.6-9.6) in the NCT group. 25.9% (22/85) of patients obtained good pathological response after neoadjuvant therapy through evaluating the surgical specimens.

Because two patients died of non-tumor-related causes and one patient had tumor rupture during surgery, we analyzed the prognostic relevance of the changes of tumor IDO1 expression and CD8+TILs status after neoadjuvant therapy in 82 patients and the median follow-up time was 33.5 months (range, 5.3-76.5). Among them, 46% (38/82) patients presented with recurrence or distant metastasis and 33% (27/82) patients died during the follow-up. Using ROC curve analysis, the optimal changes of IDO1 expression score and CD8+ TILs density cut-off value was calculated as 1.5 (sensitivity = 70.83%, specificity = 63.94%) and 29.5 (sensitivity = 58.33%, specificity = 65.57%), respectively. Patients were then divided into the H-ΔCD8 group (≥29.5), L-ΔCD8 group (<29.5), IDO1-rise group (≥1.5), and IDO1-decline group (<1.5).

### NCT Upregulates the IDO1 Expression in ESCC Cells

**Figure 1:**
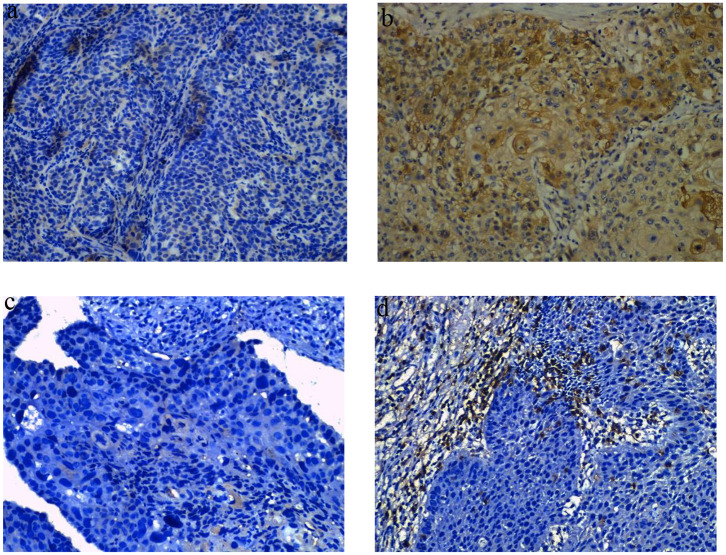
Representative images of obvious increase in tumor IDO1 expression and CD8+TILs by immunohistochemical staining in ESCC. **(a)** tumor IDO1 expression in endoscopic biopsies. **(b)** tumor IDO1 expression increased after neoadjuvant therapy in surgical specimen from the same patient. **(c)** CD8+ TILs in tumor-invasive marginal areas in endoscopic biopsies. **(d)** the density of CD8+ TILs increased after neoadjuvant therapy in tumor-invasive marginal areas from the same patient.

Tumor IDO1 expression was evaluated and compared between endoscopically biopsied tissue samples and surgical specimens. In the NCRT group, Wilcoxon rank test indicated no significant difference in tumor IDO1 expression score before and after NCRT (*p* = 0.44). However, the median IDO1 expression score significantly increased after NCT (0 vs. 15, *p* = 0.002), which was also significantly higher than that changes after NCRT (*p* = 0.02). Compared with NCRT, the tumor expression of IDO1 was more easily increased after NCT (*p* = 0.009). An example of distinct increase in tumor IDO1 expression score after neoadjuvant therapy was exhibited in Figures 1a,b.

### NCRT and NCT Upregulate the CD8+TILs Status in the TME of ESCC

We analyzed the density of CD8+TILs in the tumor-invasive margin before and after neoadjuvant treatment. The median density of CD8+TILs significantly increased from 0 to 35 after NCRT (*p* = 0.001), and also significantly increased from 5 to 30 after NCT (*p* < 0.001). There was no significant difference in density changes of CD8+TILs between the NCRT and NCT groups (*p* = 0.118). An example of density obvious increased of CD8+ TILs after neoadjuvant therapy was exhibited in Figures 1c,d.

### Correlation of Clinicopathological Features With the Changes of Tumor IDO1 Expression and CD8+TILs Status

We analyzed the relationship between clinicopathological features and the changes of tumor IDO1 expression and CD8+TILs density among 85 patients (Table 2). There were no significant differences in gender, age, tobacco use, alcohol use in the IDO1-rise and IDO1-decline groups, and also in the H-ΔCD8 and L-ΔCD8 groups. In terms of postoperative pathological response, patients in the IDO1-decline group were significantly more likely to get good response than those in the IDO1-rise group (39.1 vs. 10.3%, *p* = 0.002). Besides, IDO1-rise was also significantly associated with NCT(*p* = 0.009), pN+(*p* = 0.029), poor pathological TNM staging (III/IV) (*p* = 0.031), nerve invasion (*p* = 0.015) and vascular cancer embolus(*p* = 0.019). We also discovered that H-ΔCD8 was significantly associated with clinical tumor staging III/IV (*p* = 0.019) and NCRT (*p* = 0.028). Moreover, patients in the IDO1-rise group were more likely to have an obvious increase in the density of CD8+ TILs than patients in the IDO1-decline group after neoadjuvant treatment, although the difference was not statistically significant in all patients (51.3 vs. 32.6%, *p* = 0.081), yet there was a significant difference in patients with NCT (47.2 vs. 21.9%, *p* = 0.029).

**Table 2:** Clinicopathological features and the changes of tumor IDO1 expression and CD8+TILs density after neoadjuvant therapy.

		**IDO1-rise**	**IDO1-decline**	***p***	**L-ΔCD8**	**H-ΔCD8**	***P***
Total	85 (100%)	39 (45.9%)	46 (54.1%)		50 (58.8%)	35 (41.2%)	
Age				0.075			0.919
<70 years	70 (82.4%)	29 (74.4%)	41 (89.1%)		41 (82.0%)	29 (82.9%)	
≥70 years	15 (17.6%)	10 (25.6%)	5 (10.9%)		9 (18.0%)	6 (17.1%)	
Sex				0.973			0.536
Male	59 (69.4%)	27 (69.2%)	32 (69.6%)		36 (72.0%)	23 (65.7%)	
Female	26 (30.6%)	12 (30.8%)	14 (30.4%)		14 (28.0%)	12 (34.3%)	
Tobacco use				0.554			0.407
Yes	32 (37.6%)	16 (41.0%)	16 (34.8%)		17 (34.0%)	15 (42.9%)	
No	53 (62.4%)	23 (59.0%)	30 (65.2%)		33 (66.0%)	20 (57.1%)	
Alcohol use				0.473			0.499
Yes	40 (47.1%)	20 (51.3%)	20 (43.5%)		22 (44.0%)	18 (51.4%)	
No	45 (52.9%)	19 (48.7%)	26 (56.5%)		28 (56.0%)	17 (48.6%)	
Clinical stage				0.098			0.019
II	32 (37.6%)	11 (28.2%)	21 (45.7%)		24 (48.0%)	8 (22.9%)	
III/IV	53 (62.4%)	28 (71.8%)	25 (54.3%)		26 (52.0%)	27 (77.1%)	
TRG				0.002			0.636
I/II	63 (74.1%)	35 (89.7%)	28 (60.9%)		38 (76.0%)	25 (71.4%)	
III/IV	22 (25.9%)	4 (10.3%)	18 (39.1%)		12 (24.0%)	10 (28.6%)	
Neoadjuvant therapy				0.009			0.028
NCT	68 (80.0%)	36 (92.3%)	32 (69.6%)		44 (88.0%)	24 (68.6%)	
NCRT	17 (20.0%)	3 (7.7%)	14 (30.4%)		6 (12.0%)	11 (31.4%)	
Pathological staging				0.031			0.139
I/II	54 (63.5%)	20 (51.3%)	34 (73.9%)		35 (70.0%)	19 (54.3%)	
III/IV	31 (36.5%)	19 (48.7%)	12 (36.5%)		15 (30.0%)	16 (45.7%)	
pN staging				0.029			0.108
N0	50 (58.8%)	18 (46.2%)	32 (69.6%)		33 (66.0%)	17 (48.6%)	
N+	35 (41.2%)	21 (53.8%)	14 (30.4%)		17 (34.0%)	18 (51.4%)	
Nerve invasion				0.015			0.407
Negative	72 (84.7%)	29 (74.4%)	43 (93.5%)		41 (82.0%)	31 (88.6%)	
Positive	13 (15.3%)	10 (25.6%)	3 (6.5)		9 (18.0%)	4 (11.4%)	
Vascular cancer embolus				0.019			0.103
Negative	70 (82.4%)	28 (71.8%)	42 (91.3%)		44 (88.0%)	26 (74.3%)	
Positive	15 (17.6%)	11 (28.2%)	4 (8.7%)		6 (12.0%)	9 (25.7)	

### Upregulated Tumor IDO1 Expression After Neoadjuvant Treatment Is Associated With Poorer OS in ESCC

**Figure 2:**
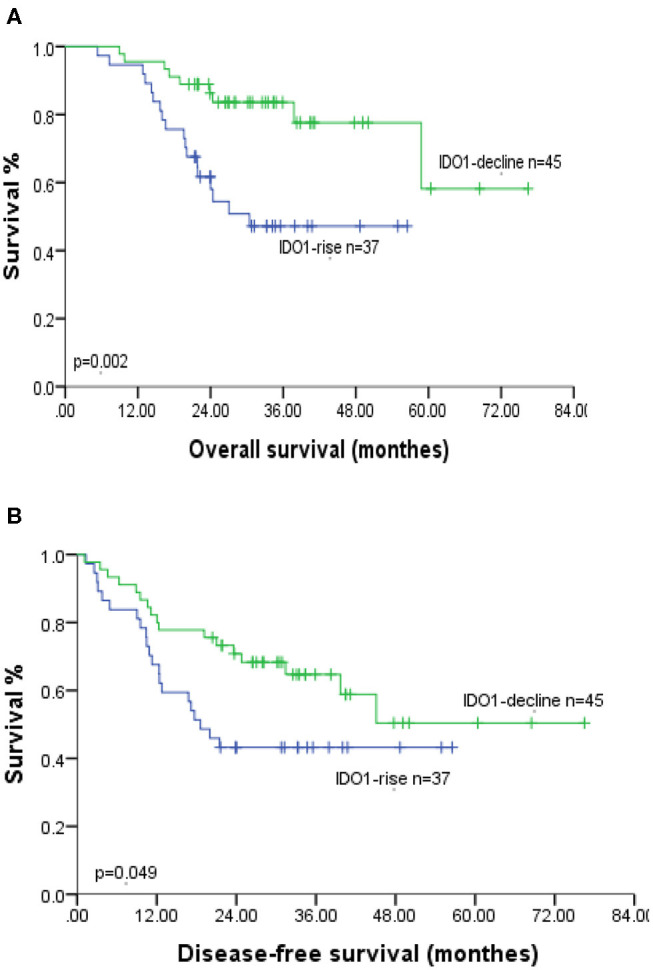
Association of OS **(A)** and DFS **(B)** with the changes of tumor IDO1 expression after neoadjuvant therapy in ESCC patients.

**Figure 3:**
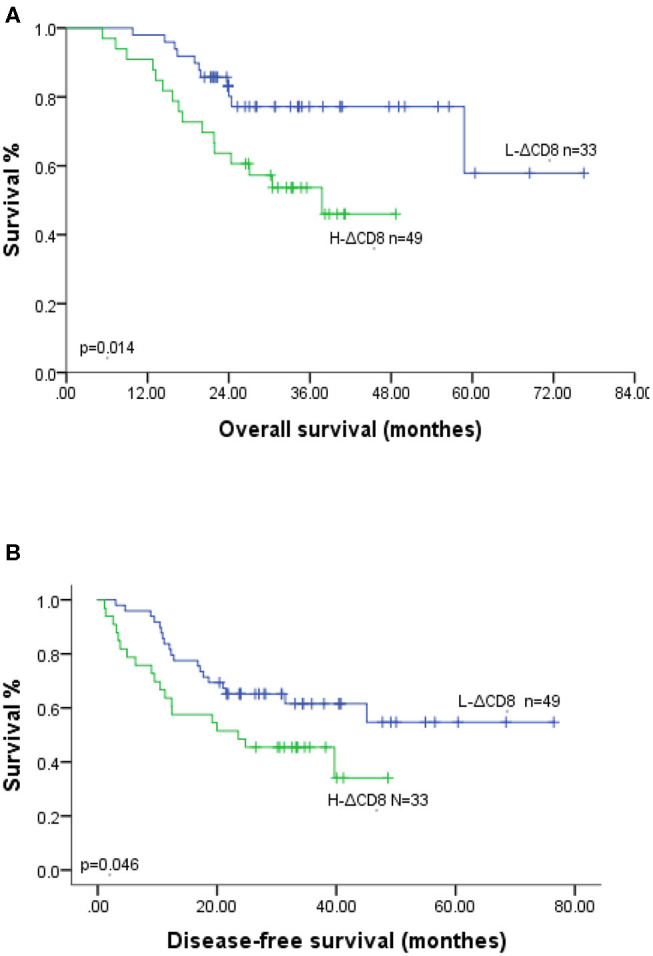
Association of OS **(A)** and DFS **(B)** with the changes of CD8+TILs after neoadjuvant therapy in ESCC patients.

Our study analyzed the prognosis in 82 ESCC patients, including 49 H-ΔCD8 patients and 45 IDO1-decline patients. In Kaplan-Meier survival analysis, patients in the IDO1-decline group achieved significantly better overall survival (OS) and disease-free survival (DFS) compared with patients in the IDO1-rise group (Figures 2A,B). Likely, patients in the L-ΔCD8 group had significantly longer OS and DFS compared with patients in the H-ΔCD8 group (Figures 3A,B). Multivariate analysis displayed that changes of tumor IDO1 expression after neoadjuvant treatment and pathological N staging were independent prognostic factors for OS, and only pathological N staging was independent prognostic factors for PFS (Tables 3,4).

**Table 3:** Cox regression analysis for OS.

	**Univariate Analysis**		**Multivariate Analysis**	
	**HR (95%CI)**	***P***	**HR (95%CI)**	***P***
Age ≥70 (vs. <70)	2.025 (0.846-4.844)	0.113	-	-
Female (vs. male)	1.335 (0.599-2.975)	0.479	-	-
Tobacco use (yes vs. no)	0.896 (0.406-1.978)	0.785	-	-
Alcohol use (yes vs. no)	1.137 (0.532-2.428)	0.741	-	-
Performance status (1 vs. 0)	4.901 (0.662-36.278)	0.120	-	-
NCRT vs. (NCT)	1.491 (0.623-3.570)	0.370	-	-
Clinical stage III/IV (vs. II)	3.580 (1.340-9.567)	0.011	1.901 (0.650-5.562)	0.241
IDO1-decline vs. IDO1-rise	0.293 (0.127-0.675)	0.004	0.416 (0.175-0.990)	0.047
H-ΔCD8 vs. L-ΔCD8	2.594 (1.176-5.724)	0.018	1.670 (0.720-3.870)	0.232
pN staging (N+ vs. N0)	4.206 (1.836-9.635)	0.001	3.275 (1.383-7.756)	0.007

**Table 4:** Cox regression analysis for DFS.

	**Univariate Analysis**		**Multivariate Analysis**	
	**HR (95%CI)**	***P***	**HR (95%CI)**	***P***
Age ≥70 (vs. <70)	1.408 (0.642-3.089)	0.393	-	-
Female (vs. male)	0.924 (0.448-1.905)	0.830	-	-
Tobacco use (yes vs. no)	1.034 (0.538-1.985)	0.921	-	-
Alcohol use (yes vs. no)	1.179 (0.623-2.233)	0.613	-	-
Performance status (1 vs. 0)	2.150 (0.659-7.014)	0.204	-	-
NCRT vs. (NCT)	1.613 (0.762-3.415)	0.211	-	-
Clinical stage III/IV (vs. II)	2.264 (1.092-4.692)	0.028	1.482 (0.674-3.257)	0.328
IDO1-decline vs. IDO1-rise	0.529 (0.279-1.006)	0.052	0.815 (0.410-1.621)	0.560
H-ΔCD8 vs. L-ΔCD8	1.898 (1.000-3.603)	0.050	1.510 (0.763-2.986)	0.237
pN staging (N+ vs. N0)	3.409 (1.754-6.625)	<0.001	2.787 (1.356-5.729)	0.005

## Discussion

In order to reduce the tumor volume and improve local control, patients with resectable ESCC usually experienced neoadjuvant therapy followed by surgery. And patients with NCRT could get better pathological complete response rate compared with patients with NCT (24, 25). Lim et al. revealed that tumor PD-L1 expression significantly increased after NCRT and significantly decreased after NRT in ESCC (18). In current study, we found that the tumor IDO1 expression significantly increased after NCT, but did not change significantly after NCRT. However, Zhou et al. discovered that the tumor expression of IDO1 also significantly increased after NCRT (26). The inconsistent results after NCRT between the two studies could be due to the different time intervals between the end of NCRT and esophagectomy, and the median time interval of the study by Zhou et al. was longer than that of our study (6.7 weeks (range, 4.1-13.1) vs. 3.1 weeks (range, 2.0-6.6), respectively), which might provide tumor cells more time to repair the damage and became resistant. Besides, our study also found that the increased tumor IDO1 expression after neoadjuvant therapy positively correlated with poor pathological response, pN+, poor pathological TNM staging (III/IV), nerve invasion and vascular tumor thrombus. As we know, the check point of IDO1 could inhibit the function of cytotoxic T cells and cause immune escape in TME and tumor-draining lymph nodes (13, 14, 27, 28). Zhou et al. demonstrated that patients with tumor IDO1 positive expression had a significantly worse pathological response in ESCC (9). In addition, higher tumor IDO1 expression was also associated with more advanced clinical stage and increased lymph node and distant metastasis in breast cancer (29–31). Muller et al. testified that IDO1 inhibitor could enhance chemotherapeutic efficacy in breast cancer of mouse models (32). Therefore, this may be one of the reasons why the pathological response of NCRT is superior to NCT. A preclinical study demonstrated that up-regulation of tumor IDO1 expression was driven by CD8+TILs in Melanoma and the infiltration of CD8+TILs was positive correlated with the expression of tumor IDO1 expression (27). In our study, the changes of tumor IDO1 expression was positively correlated with the changes of CD8+ TILs density after NCT; while no such correlation was observed in the NCRT group. Our study also manifested that the density of CD8+TILs increased significantly after NCRT compared with NCT, which might be because the addition of radiotherapy caused tumor cells to release more tumor-associated antigens and damage-associated pattern molecules to induce more numbers of CD8+TILs and immunogenic cell death (33, 34). However, the mechanisms of the changes of tumor IDO1 expression after NCRT remain to be elucidated.

Several recent studies have shown that tumor IDO1 expression was correlated with poor prognosis in esophageal cancer, endometrial cancer and glioblastoma (15, 35, 36). Conversely, other studies indicated the tumor IDO1 expression was associated with good prognosis in renal carcinoma and hepatic carcinoma (37, 38). This is the first study analyzed the relationship between the changes of tumor IDO1 expression after neoadjuvant therapy (including NCRT and NCT) and prognosis in ESCC. Our study discovered that upregulated tumor IDO1 expression after neoadjuvant therapy was independent prognostic factor for poor prognosis in ESCC patients. Up-regulation of tumor IDO1 expression after neoadjuvant therapy reflected the resistance of tumor cell to clinical treatment, which enabled us to dynamically observe the changes of TME, so as to make the long-term effect of treatment and clinical prognosis more accurate prediction. As for the changes in the density of CD8+TILs after treatment, univariate analysis revealed that patients in H-ΔCD8 group obtained shorter OS and DFS compared with patients in L-ΔCD8 group, which might be because the density of CD8+TILs significantly increased after neoadjuvant therapy was associated with poor clinical tumor staging III/IV (0.019). However, in multivariable analysis, no difference in OS and DFS was observed between the H-ΔCD8 and L-ΔCD8 groups, indicating that the body's immune function was a complicated mechanism and changed dynamically with various factors.

Recently, more and more researches have devoted to studying the combination therapy of conventional antitumor therapies and IDO1 inhibitors or PD-1/PD-L1 inhibitors, such as NCT03322566, NCT03342352, NCT02460367. And some studies indicated that tumor PD-L1 expression reduced after NCT and increased after NCRT in ESCC and rectal cancer (18, 19, 39). However, the studies on changes of tumor IDO1 expression after neoadjuvant therapy are lacked in ESCC. In current study, we found that tumor IDO1 expression was upregulated after NCT and was not observed distinct changes after NCRT in ESCC, which provided a theoretical basis for IDO1 inhibitors combined with traditional chemotherapy in ESCC. As shown in previous studies, IDO1 inhibitors could enhance the antitumor efficacy of chemotherapy regimen and the combination therapy of IDO1 inhibitor and chemotherapy might be a feasible therapeutic strategy in malignant tumor (32, 40). Existing preclinical researches have clearly confirmed that paclitaxel combined IDO1 inhibitors would restore the proliferation ability and cytotoxic response of TILs and achieved synergistic antitumor effect in breast cancer and ovarian cancer (32, 41, 42). Nevertheless, whether the efficacy of chemotherapy combined with IDO1 inhibitors as a new neoadjuvant therapy strategy is better than NCRT in ESCC remains to be further explored in future study.

Our study has several limitations. First, the sample size was relatively small, especially for the NCRT group, which was mainly due to the difficulty obtaining the paired specimens and the lack of ESCC patients who received NCRT before surgery. Second, although the PS was matched between the NCRT and NCT groups, we performed 1:4 matching of patients in the two groups, which was mainly to expand our sample size for prognostic analysis. Third, it was a retrospective clinical study and the survival prognosis may be influenced by the absence of standardized follow-up procedures. Therefore, further prospective studies or studies with large sample size are needed to validate our finding.

To summarize, our study demonstrated that tumor IDO1 expression was most likely to be affected by chemotherapy, because it increased significantly after NCT and no significant change was observed after NCRT in ESCC patients, and the increased tumor IDO1 expression after neoadjuvant therapy was associated with poor pathological response and prognosis. Further prospective studies with large sample size are warranted to confirm the influence of neoadjuvant therapy on tumor IDO1 expression in ESCC.

## Data Availability Statement

All datasets generated for this study are included in the article/supplementary material.

## Ethics Statement

The studies involving human participants were reviewed and approved by The Affiliated Cancer Hospital of Zhengzhou University. The patients/participants provided their written informed consent to participate in this study.

## Author Contributions

All authors listed have made a substantial, direct and intellectual contribution to the word, and approved it for publication.

## Conflict of Interest

The authors declare that the research was conducted in the absence of any commercial or financial relationships that could be construed as a potential conflict of interest. The reviewer Y-CZ declared a shared affiliation, with no collaboration, with one of the authors, SS, to the handling editor at the time of review.
